# Neuronal STING activation mediates inflammation-induced neurodegeneration via ferroptosis pathways in multiple sclerosis

**DOI:** 10.52601/bpr.2024.240908

**Published:** 2024-12-31

**Authors:** Weiyan Wang, Mengdi Guo, Xiao Tu, Meiling Jiang, Cun-Jin Zhang

**Affiliations:** 1 Department of Neurology, Sichuan Provincial People’s Hospital, University of Electronic Science and Technology of China, Chengdu 611731, China; 2 Department of Science and Technology, Sichuan Provincial People's Hospital, University of Electronic Science and Technology of China, Chengdu 611731, China

Multiple sclerosis (MS) is a chronic autoimmune disease of the central nervous system (CNS) which leads to severe neurological disabilities, including motor and sensory deficits, cognitive impairment, and ultimately significant reduction in the quality of life (Franklin and Simons 2022; Klotz *et al.* 2023; Marcus 2022). Although the clinically available immunosuppressive or immune-modifying therapies are capable of dampening CNS-infiltrating immune cells that drive MS relapses partially, therapeutic options for halting progressive neurodegeneration remain an unmet clinical need. Developing such therapies is limited due to an incomplete understanding of the intrinsic neuronal factors that determine neurodegeneration (Woo *et al.* 2024a). Persistent inflammation has been reported to play a role in leading to neurodegeneration and deterioration of neurological function in MS, yet the underlying mechanism is unclear. A recent study published in *Cell* by Woo *et al*. delves into the intricate processes driving inflammation-induced neurodegeneration in MS (Woo *et al.* 2024b). Their findings reveal a complex interplay among neuroinflammation, glutamate excitotoxicity, and neuronal cell death, offering a deeper insight into the pathogenic mechanisms in inflammation induced neurodegeneration.

Recent advances in neurodegeneration of multiple sclerosis have achieved some encouraging results. Some targets have already been identified, such as hnRNP A1 (Salapa *et al.* 2024), ASCL4 (Luoqian *et al.* 2022) and so on. Recently, Woo *et al*. identified neuronal STING as a central regulator of inflammation-induced neurodegeneration, providing a brand new target enriching our understanding of neuronal responses to inflammation. Researchers investigated the roles of stromal interaction molecules 1 and 2 (STIM1, STIM2) in neurodegeneration during central nervous system inflammation by generating transgenic mice with neuron-specific deletions of these genes. They found that STIM1-conditional knockout mice exhibited worse disease outcomes and increased neuronal loss in experimental autoimmune encephalomyelitis (EAE), highlighting the crucial role of STIM1 in neuronal resilience. In EAE mice and MS patients, STIM1 levels were reduced in neuronal somata but increased in injured axons, indicating a link between STIM1 deficiency and inflammation-induced neurodegeneration. *In vitro* studies demonstrated that glutamate excitotoxicity increased cytosolic calcium, leading to STIM1 translocation to the membrane and interaction with ORAI calcium release-activated calcium modulator 1 (ORAI1). Further research revealed that STING expression in neurons, induced by IFNγ or lentiviral transduction, increased vulnerability to glutamate excitotoxicity. This increased vulnerability was mitigated by STING inhibitors or by using STING1-deficient neurons, underscoring STING's role in neuronal injury.

Crucially, STING's translocation from the ER to the Golgi apparatus was essential for its activation, which induced autophagy instead of the canonical pathway, thereby exacerbating neuronal damage (Fig. 1). STING-induced autophagy led to the degradation of GPX4, a key enzyme protecting against lipid peroxidation and ferroptosis (Lee *et al.* 2023; Woo *et al.* 2024b). This degradation increased oxidative stress and neuronal cell death, which could be reversed by STING inhibitors, autophagy inhibitors, or antioxidants. Thus, STING promotes ferroptosis in inflamed neurons through autophagic GPX4 degradation. EAE animals treated with STING antagonists C176 or H151 showed reduced clinical disease scores, decreased neuronal loss, and lower levels of autophagy marker LC3 and ferroptosis marker 4-HNE, along with increased GPX4 expression. These findings highlights STING as a critical regulator of neuronal autophagy-dependent ferroptosis during neuroinflammation.

**Figure 1:**
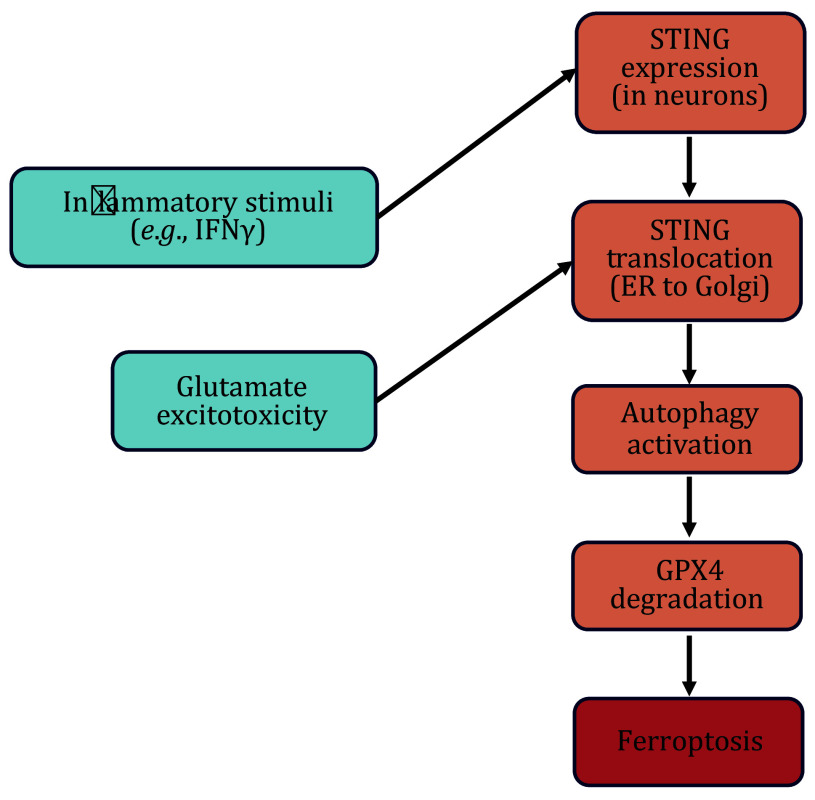
Non-canonical STING signaling pathway in neurons in MS. STING is expressed solely after inflammatory stimulation. Subsequently, glutamate excitotoxicity activates STING by facilitating its translocation from the ER to the Golgi apparatus. This activation of STING induces autophagy, leading to the degradation of GPX4 and ultimately resulting in ferroptosis

In this study, Woo *et al*. identified neuronal STING as pivotal in inflammation-driven neurodegeneration, independent of the classical cGAS pathway. IFNγ induces STING expression in CNS neurons, initially binding to STIM1 at the ER to delay activation. Glutamate later triggers STIM1 relocation, promoting STING translocation to the Golgi and subsequent activation via calcium signaling. This trafficking-mediated pathway leads to STING-induced autophagy through ULK1, culminating in GPX4 degradation and ferroptosis, crucial in MS and related neurodegenerative diseases.

In conclusion, the identification of STING as a key player in neuronal inflammatory responses by Woo *et al*. significantly contributes to our understanding of the molecular mechanisms driving neurodegeneration in MS. Beyond MS, the findings have broader implications for other neurodegenerative conditions characterized by chronic inflammation and excitotoxicity, paving the way for developing neuroprotective therapies in MS and other neurodegenerative diseases.

## Conflict of interest

Weiyan Wang, Mengdi Guo, Xiao Tu, Meiling Jiang and Cun-Jin Zhang declare that they have no conflict of interest.
